# Staphylococcal DNA Repair Is Required for Infection

**DOI:** 10.1128/mBio.02288-20

**Published:** 2020-11-17

**Authors:** Kam Pou Ha, Rebecca S. Clarke, Gyu-Lee Kim, Jane L. Brittan, Jessica E. Rowley, Despoina A. I. Mavridou, Dane Parker, Thomas B. Clarke, Angela H. Nobbs, Andrew M. Edwards

**Affiliations:** a MRC Centre for Molecular Bacteriology and Infection, Imperial College London, London, United Kingdom; b Department of Pathology, Immunology and Laboratory Medicine, Center for Immunity and Inflammation, Rutgers New Jersey Medical School, Newark, New Jersey, USA; c Bristol Dental School, University of Bristol, Bristol, United Kingdom; d Department of Molecular Biosciences, University of Texas at Austin, Austin, Texas, USA; Institut Pasteur

**Keywords:** respiratory burst, oxidative burst, DNA damage, *Enterococcus*, SOS system, *Staphylococcus*, *Streptococcus*, neutrophils

## Abstract

To cause infection, Staphylococcus aureus must withstand damage caused by host immune defenses. However, the mechanisms by which staphylococcal DNA is damaged and repaired during infection are poorly understood. Using a panel of transposon mutants, we identified the *rexBA* operon as being important for the survival of Staphylococcus aureus in whole human blood. Mutants lacking *rexB* were also attenuated for virulence in murine models of both systemic and skin infections. We then demonstrated that RexAB is a member of the AddAB family of helicase/nuclease complexes responsible for initiating the repair of DNA double-strand breaks. Using a fluorescent reporter system, we were able to show that neutrophils cause staphylococcal DNA double-strand breaks through reactive oxygen species (ROS) generated by the respiratory burst, which are repaired by RexAB, leading to the induction of the mutagenic SOS response. We found that RexAB homologues in Enterococcus faecalis and Streptococcus gordonii also promoted the survival of these pathogens in human blood, suggesting that DNA double-strand break repair is required for Gram-positive bacteria to survive in host tissues. Together, these data demonstrate that DNA is a target of host immune cells, leading to double-strand breaks, and that the repair of this damage by an AddAB-family enzyme enables the survival of Gram-positive pathogens during infection.

## Abstract (precis)

To cause infection, bacteria must survive attack by the host immune system. For many bacteria, including the major human pathogen Staphylococcus aureus, the greatest threat is posed by neutrophils. These immune cells ingest the invading organisms and try to kill them with a cocktail of chemicals that includes reactive oxygen species (ROS). The ability of S. aureus to survive this attack is crucial for the progression of infection. However, it was not clear how the ROS damaged S. aureus and how the bacterium repaired this damage. In this work, we show that ROS cause breaks in the staphylococcal DNA, which must be repaired by a two-protein complex known as RexAB; otherwise, the bacterium is killed, and it cannot sustain infection. This provides information on the type of damage that neutrophils cause S. aureus and the mechanism by which this damage is repaired, enabling infection.

## INTRODUCTION

The ability of Staphylococcus aureus to maintain the integrity of its DNA in the face of reactive oxygen and nitrogen species produced by host immune defenses is crucial for the establishment of infection. However, despite the importance of DNA repair for staphylococcal survival in the host, little is known about the processes responsible, with most functions based on inferences from work done with the model organisms Bacillus subtilis or Escherichia coli (1, 2).

This is important because S. aureus is responsible for a raft of serious invasive infections, including bacteremia, infective endocarditis, and osteomyelitis (3). Despite a potent immune response, many infections become chronic or recurrent (4), implying either that S. aureus does not experience DNA damage during infection or that it has efficient mechanisms for damage repair.

The entry of S. aureus into normally sterile tissues triggers the infiltration of neutrophils to control infection (5–7). Neutrophils phagocytose S. aureus and expose the bacterium to a cocktail of antimicrobial peptides and proteases (8–10), reactive nitrogen species, and reactive oxygen species (ROS) that are generated by the respiratory burst (also known as the oxidative burst) (11–16). While the contribution of each ROS to bactericidal activity is the subject of investigation, there is compelling evidence that the respiratory burst is crucial for the control of S. aureus infection (13, 16, 17). For example, individuals with chronic granulomatous disease (CGD) are particularly prone to staphylococcal infections because their neutrophils are defective for the respiratory burst (6, 18). In keeping with this, S. aureus survives better in mice defective for the respiratory burst than in wild-type animals, while the treatment of human neutrophils with an inhibitor of the respiratory burst increased staphylococcal survival relative to untreated immune cells (7–9, 13, 19). However, even when the respiratory burst is functional, there is evidence that some S. aureus cells can survive in neutrophils, which contributes to the progression of infection (8, 9).

Despite the importance of the respiratory burst in combating staphylococcal infection, relatively little is known about how it kills the pathogen. Studies with single oxidants such as H_2_O_2_ indicate that the molecular targets of the respiratory burst are broad and include proteins, lipids, and DNA (20). To survive this damage, S. aureus employs several stress response regulators and repair systems. For example, previous work has shown that DNA damage caused by S. aureus exposure to H_2_O_2_ leads to the initiation of the DNA repair SOS response (21), which facilitates the excision of damaged bases or the repair of double-strand breaks (DSBs) by homologous recombination (22). However, H_2_O_2_ is a suboptimal model for the ROS produced by the respiratory burst because it is typically used at concentrations that exceed those generated by the respiratory burst (23). Therefore, it is unclear whether staphylococcal DNA is damaged by neutrophil-generated ROS, what the nature of this damage is, how it is repaired by S. aureus, and the impact of this damage on infection.

To address this gap in our knowledge, we examined mutants defective for DNA repair and found that a member of the AddAB helicase/nuclease family of enzymes was required for staphylococcal survival in blood and murine models of systemic and skin infections. We also demonstrated that this complex is required for the repair of DNA double-strand breaks caused by ROS produced by the respiratory burst of neutrophils, which leads to the induction of the mutagenic SOS response. Similar complexes were required for the survival of the infective endocarditis pathogens Streptococcus gordonii and Enterococcus faecalis in human blood, demonstrating that DNA damage repair is an important mechanism by which Gram-positive pathogens withstand host defenses.

## RESULTS

### RexAB is required for staphylococcal survival in host tissues.

To determine whether DNA damage occurs under infection conditions, we assembled a panel of S. aureus mutants from the ordered Nebraska transposon library produced by the Network on Antimicrobial Resistance in S. aureus (NARSA) (24). Each mutant was defective for a different protein associated with DNA repair, which would enable the nature of any damage to be identified. We then assessed the contribution of each repair protein to bacterial survival in the host by measuring CFU counts of mutants in an *ex vivo* whole human blood model of infection. Previous characterizations of this model system by our group and others have shown that S. aureus is rapidly phagocytosed by neutrophils and exposed to ROS and other killing mechanisms (25–27).

**FIG 1:**
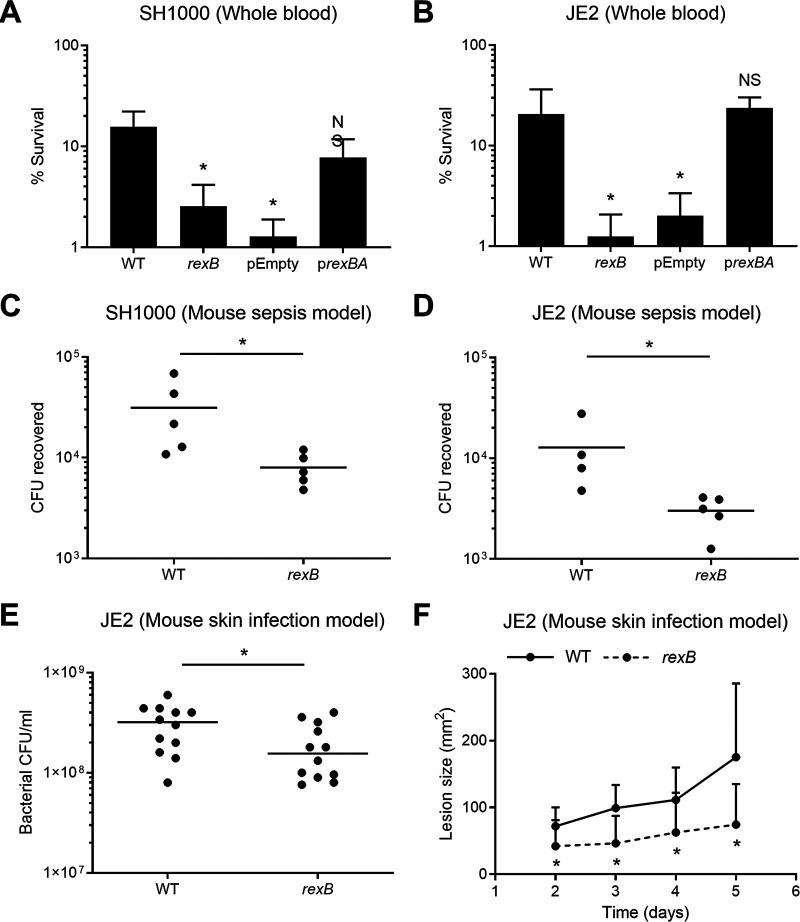
Survival of the *rexB* mutant is significantly reduced in human blood and *in vivo* murine infection models. (A and B) Survival of the S. aureus wild type (WT), the *rexB* mutant, the empty vector (pEmpty), and the complemented mutant (p*rexBA*) in the SH1000 (A) and JE2 (B) backgrounds after 6 h of incubation in whole human blood. Empty vectors and complemented mutants were supplemented with 100 ng/ml AHT (anhydrotetracycline) to induce *rexBA* expression (*n* ≥ 3). (C and D) Numbers of CFU recovered from the peritoneal cavities of mice at 6 h postinfection (each circle represents a single mouse; *n* = 4 to 5). (E) CFU per milliliter recovered from mouse skin biopsy specimens at 5 days postinfection (each circle represents a single mouse; *n* = 12). (F) Sizes of skin lesions observed on mice at up to 5 days postinfection (*n* = 12). Data in panels A and B were analyzed by one-way ANOVA with Tukey’s *post hoc* test relative to the WT (*, *P* < 0.05). Data in panels C to F were analyzed by a Mann-Whitney test (*, *P* < 0.05). NS, not significant.

Wild-type (WT) S. aureus JE2 survived relatively well in human blood, with >60% of the inoculum being viable after 2 h (see Fig. S1 in the supplemental material). In contrast, the survival of mutants defective for *rexA* or *rexB* was <5% of the inoculum (Fig. S1). Since the transposon insertion in *rexB* contains a terminator sequence that prevents the transcription of *rexA*, the second gene in the operon, *rexB* mutants are defective for both *rexB* and *rexA* and are effectively *rexBA* mutants (24). Therefore, we confirmed the importance of RexAB for the survival of two distinct strains (JE2 and SH1000) in blood by complementation of the *rexB* mutants with a plasmid containing the *rexBA* operon (p*rexBA*), which restored bacterial survival to wild-type levels (Fig. 1A and B). In contrast, the survival of *rexB* mutants transformed with the vector alone (pEmpty) was not changed from that of the mutant (Fig. 1A and B).

10.1128/mBio.02288-20.1 FIG S1 Mutants lacking functional copies of *rexA* and *rexB* have the lowest survival of a range of DNA repair mutants in whole human blood. Survival of the S. aureus JE2 wild type and a number of DNA repair mutants from the Nebraska transposon mutant library in whole human blood after 2 h of incubation (*n* ≥ 4) is shown. SOS1-7 refers to genes of unknown function within the S. aureus SOS regulon. (SOS1, SAUSA300_0365/SACOL0436; SOS2, SAUSA300_1238/SACOL1330; SOS3, SAUSA300_1903/SACOL1986; SOS4, SAUSA300_1915/SACOL1999; SOS5, SAUSA300_2129/SACOL2160; SOS6, SAUSA300_2130/SACOL2161; SOS7, SAUSA300_2131/SACOL2162). Mutants with lower percentages of survival than the JE2 WT (represented by the dotted line) are shown in white. Error bars represent standard deviations of the means. Download FIG S1, TIF file, 0.3 MB. Copyright © 2020 Ha et al. 2020 Ha et al. This content is distributed under the terms of the Creative Commons Attribution 4.0 International license.

Having shown that RexAB contributed to staphylococcal tolerance of neutrophil-mediated killing in human blood, we then tested its role in staphylococcal survival *in vivo* using a murine model of systemic infection. Mice were infected via the peritoneal cavity, which results in the recruitment of neutrophils within 2 h, with wild-type or *rexB* mutant strains of S. aureus SH1000 or JE2 (28). After 6 h, the mice were sacrificed, and the peritoneal cavity was washed with phosphate-buffered saline (PBS) to recover bacteria, which were quantified by CFU counts. This revealed that *rexB* mutants in both genetic backgrounds were significantly attenuated for survival *in vivo*, with ∼5-fold-lower CFU counts than those of the respective wild-type bacteria (*P* ≤ 0.05), confirming that RexAB contributes to staphylococcal resistance to host immune defenses and the progression of systemic infection (Fig. 1C and D).

Because S. aureus causes many different types of infection, and the associated immune responses might vary, we next assessed the survival of wild-type strain JE2 and the *rexB* mutant in a murine skin infection model. Mice were infected via subcutaneous injection, and infection was allowed to progress for 5 days before CFU counts at inoculation sites were determined. This revealed that wild-type bacteria were present at 2- to 3-fold-higher levels than the *rexB* mutant (*P* ≤ 0.05) (Fig. 1E). We also measured the sizes of the skin lesions generated by injected S. aureus. Wild-type JE2 caused a lesion that progressively increased in size over time (Fig. 1F). In contrast, the lesion caused by the *rexB* mutant did not increase after day 2 and was significantly smaller than that caused by the wild type for days 2, 3, 4, and 5 (Fig. 1F; Fig. S2). This indicated that RexAB was also required for skin infection progression.

10.1128/mBio.02288-20.2 FIG S2 Photographs of skin lesions caused by the S. aureus wild type and the *rexB* mutant. Representative images of skin lesions from WT- and *rexB* mutant-infected mice at 5 days postinfection are shown. Download FIG S2, TIF file, 0.4 MB. Copyright © 2020 Ha et al. 2020 Ha et al. This content is distributed under the terms of the Creative Commons Attribution 4.0 International license.

To understand whether the reduced CFU counts of the *rexB* mutants relative to the wild type in animal models were due to differences in growth rates or reduced virulence factor production, we measured bacterial growth, hemolysin production, staphyloxanthin levels, and catalase levels across JE2 and SH1000 wild-type and mutant strains. For both JE2 and SH1000, the *rexB* mutant replicated at a slightly lower rate than the wild type, but there were no significant differences in the production of hemolysin, staphyloxanthin, or catalase (Fig. S3). Taken together, these findings demonstrate that RexAB significantly promotes staphylococcal survival in host tissues and is required for infection progression.

10.1128/mBio.02288-20.3 FIG S3 Phenotypic properties of the wild type and the *rexB* mutant. (A) Growth curves of the WT, the *rexB* mutant, the empty vector (pEmpty), and the complemented mutant (p*rexBA*) in TSB in the SH1000 and JE2 backgrounds. TSB was supplemented with 100 ng/ml of AHT for pEmpty and p*rexBA* to control the expression of *rexAB* (*n* = 3). (B to D) Graphs showing toxin production (B), staphyloxanthin production (C), and catalase activity (D) of the WT, the *rexB* mutant, the empty vector (pEmpty), and the complemented mutant (p*rexBA*) in the JE2 and SH1000 backgrounds. Cultures of pEmpty and p*rexBA* were grown in the presence of 100 ng/ml of AHT (*n* ≥ 3). Toxin production was determined by measuring hemolytic activity as an indicator of *agr* operon expression. Catalase activity measured the degradation of H_2_O_2_ after 15 min of incubation with S. aureus cultures, which detected significance for the SH1000 WT versus the *rexB* mutant, but this was not seen for the SH1000 WT versus pEmpty or in JE2. Data were analyzed by one-way ANOVA with Tukey’s *post hoc* test (*, *P* < 0.05). Where shown, error bars represent standard deviations of the means. Error bars were omitted from panels A and B for clarity. Download FIG S3, TIF file, 0.6 MB. Copyright © 2020 Ha et al. 2020 Ha et al. This content is distributed under the terms of the Creative Commons Attribution 4.0 International license.

### RexAB is a member of the AddAB family of ATP-dependent helicase/nucleases.

The *rexA* and *rexB* genes form a two-gene operon (*rexBA*) on the staphylococcal chromosome and are proposed to encode an AddAB helicase/nuclease enzyme on the basis of sequence homology (29–32). However, this had not been demonstrated experimentally.

Our initial *in silico* structural analysis of the predicted *rexBA* gene products supported predictions that this operon encodes an AddAB-type ATP-dependent helicase/nuclease that contributes to the processing and repair of DNA DSBs (Fig. S4). AddAB enzymes process DSBs to generate a 3′ single-stranded DNA (ssDNA) overhang that is necessary for RecA-mediated homologous recombination (33). In support of the structural predictions, phenotypic testing of *rexBA* mutants showed that they were >8-fold more susceptible than wild-type strains to the DNA-damaging antibiotics ciprofloxacin and mitomycin C, both of which cause DNA DSBs (34, 35) (Fig. S5).

10.1128/mBio.02288-20.4 FIG S4 Structural analysis of S. aureus RexAB. (A) Conserved functional motifs in S. aureus RexAB and AddAB homologues. A multiple-sequence alignment of conserved motifs from AddA and AddB is shown. AddAB motifs associated with enzyme function were well conserved across the bacterial species examined, with the majority of residues being either identical or similar (140 out of 190 in total; 74%). Identical residues are indicated by “*”, highly similar residues are indicated by “:”, and less similar residues are indicated by “.”. Residue positions are indicated on the right-hand side. The alignment was generated using Clustal Omega (78), with protein sequences obtained from the NCBI Protein Database (79). Saur, S. aureus; Bsub, Bacillus subtilis; Spyo, Streptococcus pyogenes; Spne, Streptococcus pneumoniae; Efae, Enterococcus faecalis. (B) Predicted models of S. aureus RexA and RexB superimposed onto individual AddA and AddB subunits of the B. subtilis AddAB crystal structure. For S. aureus RexA, 1,110 residues (91% of the sequence) were modeled with 100% confidence to B. subtilis AddA, and for S. aureus RexB, 1,115 residues (96%) were modeled with 100% confidence to B. subtilis AddB. Shown is a ribbon representation of S. aureus and B. subtilis proteins individually and superimposed. Individual protein structures are colored from blue to red from the N to C termini. When superimposed, S. aureus proteins are shown in blue, and B. subtilis proteins are shown in orange. S. aureus RexA and RexB predicted three-dimensional (3D) models were generated using Phyre2 (84). Protein structures were visualized using PyMOL. The structure under PDB accession number 3U4Q was used for the B. subtilis AddAB structure (85, 86). Download FIG S4, TIF file, 0.6 MB. Copyright © 2020 Ha et al. 2020 Ha et al. This content is distributed under the terms of the Creative Commons Attribution 4.0 International license.

10.1128/mBio.02288-20.5 FIG S5 The *rexBA* mutants are more susceptible than the wild type to antibiotics that cause DNA DSBs. Shown are the MICs of the S. aureus WT, the *rexB* mutant, the empty vector (pEmpty), and the complemented mutant (p*rexBA*) in the SH1000 and JE2 backgrounds for ciprofloxacin (A and B) and mitomycin C (C and D). Empty vectors and complemented mutants were supplemented with 100 ng ml^−1^ AHT to induce *rexBA* expression (*n* = 3; the median MIC is shown). Error bars represent standard deviations of the means. Download FIG S5, TIF file, 0.3 MB. Copyright © 2020 Ha et al. 2020 Ha et al. This content is distributed under the terms of the Creative Commons Attribution 4.0 International license.

**FIG 2:**
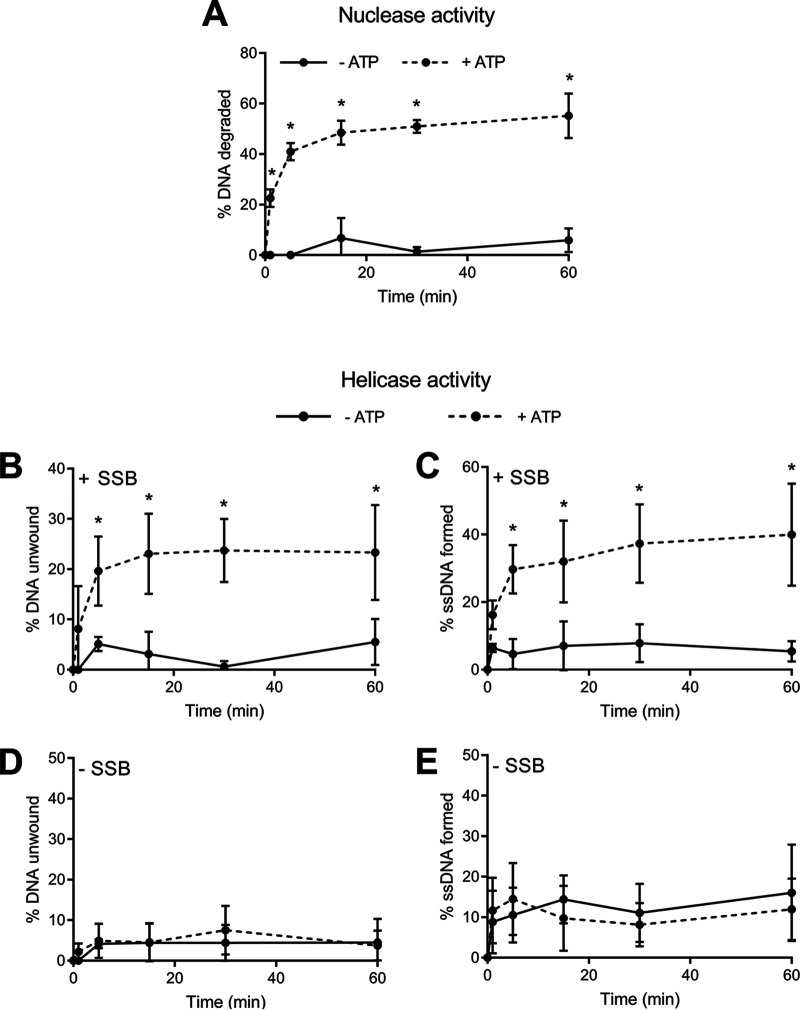
RexAB possesses ATP-dependent nuclease and helicase activities. (A) Nuclease activity of recombinant S. aureus RexAB protein in the presence or absence of ATP (*n* = 4). (B to E) Helicase activity of recombinant S. aureus RexAB protein in the presence or absence of ATP, determined by measuring the unwinding of dsDNA and the formation of ssDNA. (B and C) Single-stranded DNA binding protein (SSB) was added to prevent the reannealing of DNA (*n* = 4). (D and E) Equivalent assays were performed without SSB to confirm helicase activity (*n* = 4). Data in panels A to C were analyzed by two-way ANOVA with Sidak’s *post hoc* test comparing activities without versus those with ATP (*, *P* < 0.05). Error bars represent standard deviations of the means.

To confirm the ATP-dependent helicase/nuclease activity of the S. aureus RexAB complex, recombinant RexAB protein was generated, and the helicase and nuclease activities were measured over 1 h. Nuclease activity assays were performed under conditions of high free Mg^2+^, which has been previously shown to activate nuclease activity in AddAB enzymes (36). We found that DNA was degraded over time by the recombinant complex in the presence of ATP, whereas this degradation was minimal in its absence, demonstrating that RexAB has ATP-dependent nuclease activity (Fig. 2A).

We measured helicase activity under conditions of low free Mg^2+^ levels and observed a loss of double-stranded DNA (dsDNA) concomitant with increased ssDNA formation over time (Fig. 2B and C). These experiments were repeated in the absence of single-stranded DNA binding protein (SSB), which is required to prevent the reannealing of DNA. In the absence of SSB, no DNA unwinding or ssDNA formation was observed, demonstrating that RexAB has ATP-dependent helicase activity (Fig. 2D and E).

Combined, these results confirm that RexAB is a functional member of the AddAB family of DNA repair complexes. In turn, this demonstrates that DNA DSBs occur during staphylococcal infection and must be repaired for bacterial survival.

### RexAB enables staphylococcal tolerance of ROS produced by the respiratory burst.

Having confirmed that DNA DSBs occur in S. aureus during infection, we wanted to determine whether this was due to ROS produced by the respiratory burst of neutrophils. Therefore, we incubated wild-type and *rexB* mutant strains in whole human blood in the presence of diphenyleneiodonium chloride (DPI) to block the NADPH oxidase-generated respiratory burst or dimethyl sulfoxide (DMSO) alone as a solvent control.

**FIG 3:**
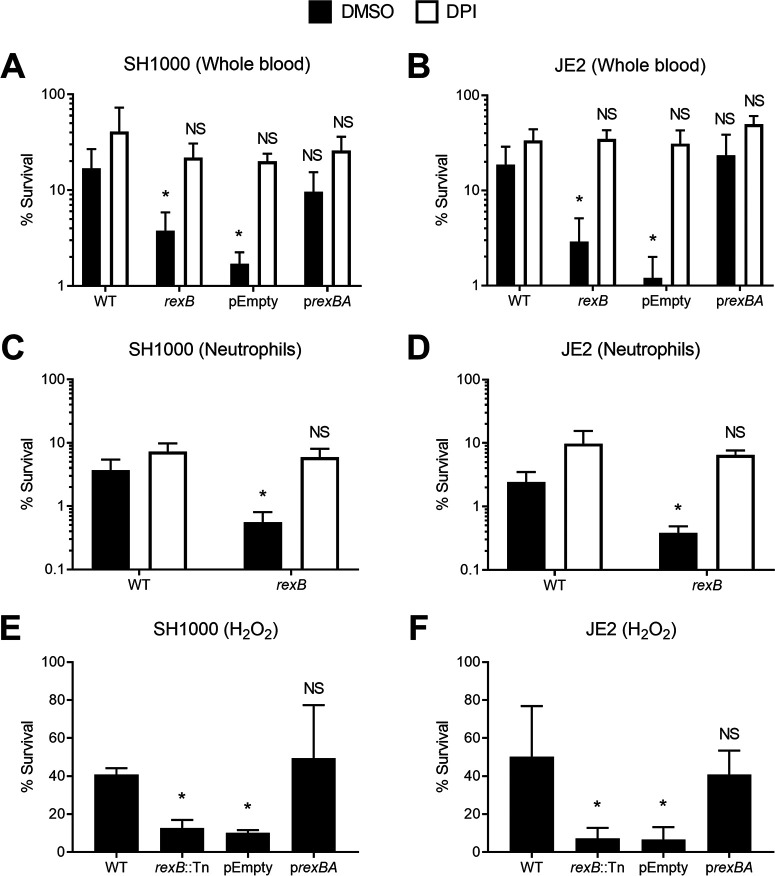
RexAB protects S. aureus from ROS produced by the respiratory burst of neutrophils and when exposed to H_2_O_2_. (A and B) Survival of the S. aureus WT, the *rexB* mutant, the empty vector (pEmpty), and the complemented mutant (p*rexBA*) in the SH1000 (A) and JE2 (B) backgrounds in whole human blood after 6 h of incubation in the presence of the respiratory burst inhibitor DPI or an identical volume of the DMSO solvent alone. Empty vectors and complemented mutants were supplemented with 100 ng/ml AHT to control *rexBA* expression (*n* = 3). (C and D) Survival of S. aureus WT and *rexB* mutant strains incubated with purified human neutrophils for 3 h (*n* = 4). (E and F) Survival of the S. aureus WT, the *rexB* mutant, the empty vector (pEmpty), and the complemented mutant (p*rexBA*) after 1 h of incubation with 10 mM H_2_O_2_. Empty vectors and complemented mutants were supplemented with 100 ng/μl AHT to induce *rexBA* expression (*n* = 3). Data in panels A and B were analyzed by one-way ANOVA with Tukey’s *post hoc* test relative to the WT (*, *P* < 0.05). Data in panels C and D were analyzed by Student’s *t* test relative to the WT (*, *P* < 0.05). Error bars represent standard deviations of the means. Data in panels E and F were analyzed by one-way ANOVA with Dunnett’s *post hoc* test relative to the WT (*, *P* < 0.05).

As shown in Fig. 1A and B, the survival of *rexB* mutants in whole human blood was significantly reduced relative to that of wild-type bacteria (Fig. 3A and B). However, the presence of DPI promoted the survival of *rexB* mutants to wild-type levels, indicating that the survival deficit observed for bacteria lacking RexAB was due to increased sensitivity to ROS produced by the respiratory burst (Fig. 3A and B). To ensure that the killing of *rexB* mutants in blood was due to neutrophils, S. aureus strains were incubated with purified human neutrophils, and survival was measured via CFU counts. Similar to whole blood, *rexB* mutants were more susceptible to neutrophil-mediated killing than the wild type, but the presence of DPI restored the survival of the *rexB* mutants to wild-type levels (Fig. 3C and D). These data strongly indicated that RexAB contributed to staphylococcal survival of DNA damage caused by ROS produced by the respiratory burst.

To confirm that *rexB* mutants were more susceptible to oxidative damage, we measured the survival of S. aureus strains in H_2_O_2_, which is one of several different ROS produced in the respiratory burst (6). As observed for whole human blood and purified neutrophils, *rexB* mutants were more susceptible to H_2_O_2_ than the wild-type or complemented strains (Fig. 3E and F).

Together, these data demonstrate that neutrophils cause DNA DSBs in S. aureus via ROS produced by the respiratory burst. This damage must be repaired by RexAB to enable staphylococcal survival in the host.

### RexAB is required for induction of the SOS response during exposure to ROS produced by the respiratory burst.

The processing of DNA DSBs by AddAB proteins leads to the generation of a 3′ overhang. This results in the formation of a RecA filament, which triggers the SOS response, a multicomponent DNA repair mechanism that mediates the repair of the DNA DSB (1, 22). However, the induction of the SOS response also leads to a transient increase in the mutation rate, which promotes the emergence of mutants with resistance to antibiotics or host-adapted phenotypes such as small-colony variants (2, 37).

**FIG 4:**
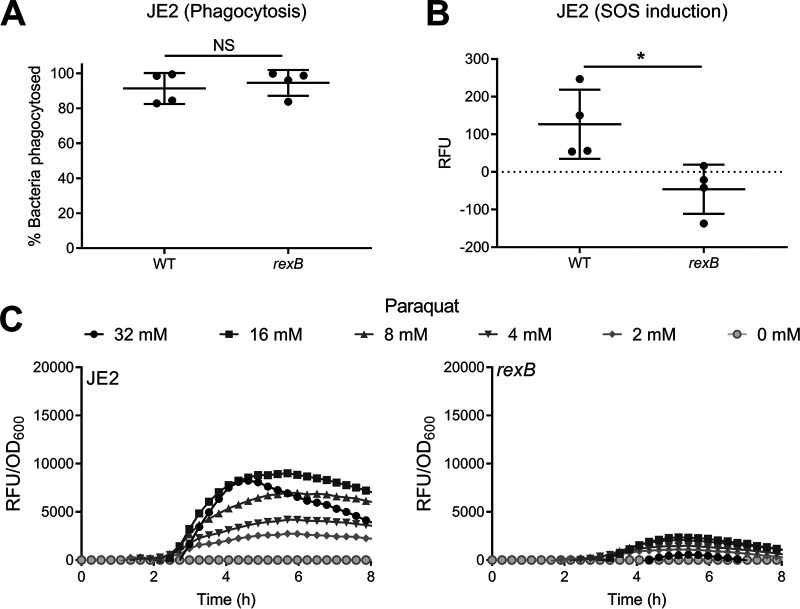
RexAB is required for induction of the SOS response during exposure to ROS produced by the respiratory burst of neutrophils and when exposed to the ROS generator paraquat. (A and B) Phagocytosis (both ingested and surface-bound bacteria [39]) of JE2 wild-type and *rexB* mutant *recA-gfp* reporter strains by neutrophils (A) and the resulting induction of the SOS response measured by GFP expression (RFU) after 30 min of exposure (*n* = 4) (B). (C) Induction of the SOS response of JE2 wild-type and *rexB* mutant strains as measured by GFP expression upon exposure to sublethal concentrations of paraquat. GFP fluorescence was normalized by the OD_600_ to determine the induction of SOS relative to the cell density (*n* = 4). OD_600_ measurements alone are shown in Fig. S5 in the supplemental material. Data in panels A and B were analyzed by a Mann-Whitney test (*, *P* < 0.05). Where shown, error bars represent standard deviations of the means. Error bars were omitted from panels C and D for clarity.

Therefore, we tested whether ROS produced by the respiratory burst triggers the SOS response and whether this was dependent upon RexAB. To do this, we used a P*recA-gfp* reporter construct and validated it by showing dose-dependent activity with mitomycin C, a well-established trigger of the SOS response (38) (Fig. S6). We then incubated tetramethyl rhodamine isothiocyanate (TRITC)-labeled S. aureus JE2 wild type and *rexB* mutant strains containing the reporter with neutrophils for 30 min and used flow cytometry to measure phagocytosis and reporter activity (green fluorescent protein [GFP] fluorescence) (Fig. S7) (39). As reported previously, >95% of S. aureus cells were associated with neutrophils within 30 min (Fig. 4A) (26). Also, by 30 min, there was an increase in the GFP signal from wild-type S. aureus relative to the start of the assay (Fig. 4B). In contrast, there was no increase in the GFP signal from the *rexB* mutant (Fig. 4B), indicating that neutrophils trigger the SOS response in S. aureus via DNA processing by RexAB.

10.1128/mBio.02288-20.6 FIG S6 Mitomycin C causes significantly greater dose-dependent induction of SOS in the WT than in the *rexB* mutant. The induction of the SOS response of the JE2 WT (A) and the *rexB* mutant (B) was measured by GFP expression upon exposure to various sublethal concentrations of mitomycin C. Concentrations of antibiotics are labeled as multiples of the MIC of the WT strain (× MIC). RFU data are normalized to the OD_600_ data to generate *recA* expression relative to the cell density (*n* = 3). Error bars were omitted for clarity. Download FIG S6, TIF file, 0.5 MB. Copyright © 2020 Ha et al. 2020 Ha et al. This content is distributed under the terms of the Creative Commons Attribution 4.0 International license.

10.1128/mBio.02288-20.7 FIG S7 Gating strategy for flow cytometry data comparing WT and *rexB* mutant *recA-gfp* reporter strains during incubation with neutrophils. (A and B) Bacterium-only control gated by forward and side scatter and subsequently by TRITC labeling. (C) Neutrophil-only control gated by forward and side scatter. (D) No TRITC labeling present in the neutrophil sample. (E and F) Gating strategy used for each sample and time point. (E) Neutrophils and bacteria were gated by forward and side scatter. (F) Neutrophils were plotted using 586_16 YG-A for TRITC staining, which revealed two distinct populations: neutrophils only (no TRITC detection) and neutrophils containing phagocytosed and surface-bound TRITC-labeled bacteria. The numbers of phagocytosed/surface-attached bacteria were compared to the total number of bacteria to calculate the percentage of bacteria associated with neutrophils at each time point. To quantify DNA damage, neutrophils containing phagocytosed or surface-attached TRITC-labeled bacteria were isolated, and the mean GFP value was determined at each time point. Relative GFP detection was used to compare the induction of the SOS response between the JE2 WT and *rexB* mutant strains. Download FIG S7, TIF file, 0.4 MB. Copyright © 2020 Ha et al. 2020 Ha et al. This content is distributed under the terms of the Creative Commons Attribution 4.0 International license.

To further explore the requirement for RexAB for the induction of the SOS response during exposure to oxidative stress, wild-type and *rexB* mutant bacteria were incubated with various subinhibitory doses of paraquat, which results in the generation of endogenous superoxide, which dismutates to H_2_O_2_. For wild-type bacteria, there was a clear dose-dependent increase in GFP-mediated fluorescence, indicative of SOS induction (Fig. 4C). For the *rexB* mutant, while there also appeared to be a dose-dependent induction of the SOS response, it was at considerably lower levels than those seen for the wild type (Fig. 4C). Therefore, the induction of SOS in response to oxidative stress is almost entirely dependent upon RexAB-mediated processing of DNA DSBs.

### RexAB is required for survival of streptococci and enterococci in human blood.

Since homologues of RexAB are present in most Gram-positive bacteria (31–33), we next tested whether this repair complex contributes to the survival of other bacteria exposed to neutrophils. Like S. aureus, Enterococcus faecalis and Streptococcus gordonii are frequent causes of infective endocarditis, which brings these species into close contact with neutrophils (40, 41). Therefore, *rexBA* was deleted in representative strains of each species, and their sensitivity to the microbicidal activity of neutrophils was determined using the *ex vivo* whole human blood model. To confirm that RexAB mediates the repair of DNA DSBs in both bacteria, we also assessed their susceptibility to the antibiotics ciprofloxacin, which causes DNA DSBs (42), and gentamicin, which targets protein synthesis and thus acted as a negative control (43). For both S. gordonii and E. faecalis, the Δ*rexBA* mutants were significantly more susceptible to ciprofloxacin than the wild type, confirming that RexAB in these bacteria contributes to DNA DSB repair (Table S1). In contrast, both the wild type and the Δ*rexBA* mutants were equally susceptible to the antibiotic gentamicin (Table S1).

10.1128/mBio.02288-20.8 TABLE S1 MICs of selected antibiotics for the wild type and Δ*rexAB* mutants of S. gordonii DL1 and E. faecalis OG1X. For each strain, the MIC (micrograms per milliliter) of the indicated antibiotics is shown. Data represent the medians from 3 independent experiments. Download Table S1, DOCX file, 0.01 MB. Copyright © 2020 Ha et al. 2020 Ha et al. This content is distributed under the terms of the Creative Commons Attribution 4.0 International license.

**FIG 5:**
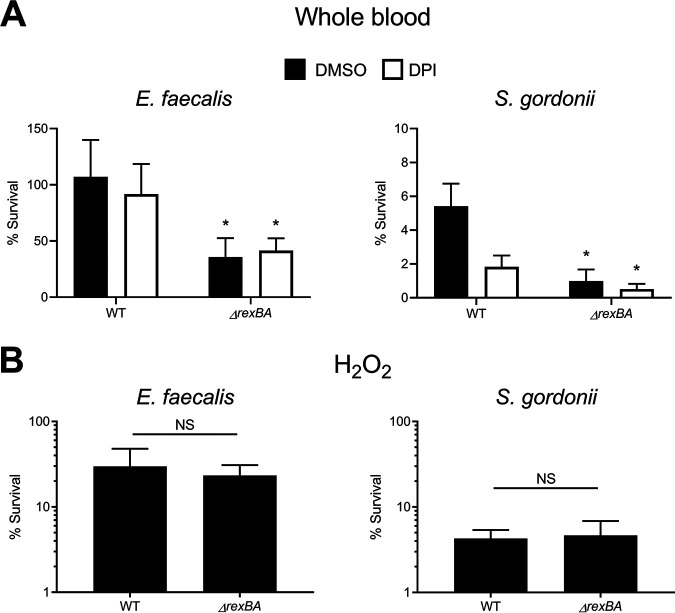
RexAB protects Enterococcus faecalis and Streptococcus gordonii from host defenses in blood. (A) Survival of E. faecalis and S. gordonii wild-type and Δ*rexBA* mutant strains in whole human blood after 6 h in the presence of the respiratory burst inhibitor DPI or an identical volume of the DMSO solvent alone (*n* = 3). (B) Survival of E. faecalis and S. gordonii wild-type and Δ*rexBA* mutant strains exposed to H_2_O_2_ for 1 h (*n* = 3). Data in panels A and B were analyzed by Student’s *t* test comparing the Δ*rexBA* mutant versus the WT (*, *P* < 0.05). Error bars represent standard deviations of the means.

Wild-type E. faecalis survived at high levels in human blood, with ∼100% of the inoculum remaining viable during the full 6-h duration of the assay, but the loss of *rexBA* reduced enterococcal survival by ∼50% (Fig. 5A). This indicated that E. faecalis suffers DNA damage that results in DSBs while in blood, but it can be tolerated via DNA repair (Fig. 5A). However, in contrast to S. aureus, DNA damage in E. faecalis was not due to ROS produced by the respiratory burst since the survival of both wild-type and *rexB* mutant bacteria in blood was unaffected by the presence of DPI (Fig. 5A).

S. gordonii was much more susceptible to host defenses in blood than E. faecalis, with ∼5% of wild-type bacteria remaining viable after 6 h (Fig. 5A). However, the survival of the *rexBA* mutant was still reduced relative to the wild type, with ∼1% of streptococci surviving after 6 h, indicating that DNA DSB repair also contributes to the survival of S. gordonii in blood (Fig. 5A). As for E. faecalis, the presence of DPI did not increase the survival of S. gordonii in blood, indicating that DNA damage was not due to ROS produced by the respiratory burst in either bacterium. In support of these findings, the *rexBA* mutants of both S. gordonii and E. faecalis were no more susceptible to H_2_O_2_ than wild-type bacteria (Fig. 5B).

Therefore, as for S. aureus, neutrophils damage DNA of both S. gordonii and E. faecalis, the repair of which by RexAB promotes bacterial survival. However, in contrast to S. aureus, ROS generated by the neutrophil respiratory burst do not appear to contribute to DNA damage in these bacteria.

## DISCUSSION

Neutrophils are an essential host defense against S. aureus and many other bacterial pathogens. However, our understanding of the mechanisms by which these immune cells kill staphylococci is limited. The data presented here demonstrate that neutrophils cause DNA damage in S. aureus via ROS produced by the respiratory burst, the repair of which requires the RexAB complex and leads to the induction of the SOS response. We also confirmed that RexAB is a member of the AddAB helicase/nuclease family of enzymes involved in the processing of DNA DSBs for repair via homologous recombination. Thus, ROS generated by neutrophils cause DNA DSBs in S. aureus, which are lethal if not repaired. This provides new information on both the target of the ROS generated by the respiratory burst and the mechanisms by which S. aureus repairs and survives this damage.

The importance of RexAB for staphylococcal survival during infection was demonstrated in murine models of both systemic and skin infections as well as an *ex vivo* whole human blood model of bacteremia. Since S. aureus is a frequent cause of both superficial and systemic infections (3), these findings confirm the importance of DNA repair for staphylococcal survival in relevant but distinct host tissues.

Previous work demonstrated the requirement of DNA DSB repair for the survival of Gram-negative pathogens *in vivo*. For example, AddAB was shown to be required for the infection of chickens and mice by Campylobacter jejuni and Helicobacter pylori, respectively (44, 45). Furthermore, the virulence of Salmonella enterica in a murine model of bacteremia was dependent upon the RecBCD DNA DSB repair complex (46). However, the importance of such systems for Gram-positive bacteria was unclear.

DNA damage occurred within 30 min of phagocytosis of S. aureus by neutrophils, which corresponds to the time at which ROS are maximally generated in these immune cells (47, 48). The finding that the respiratory burst leads to DNA DSBs in S. aureus is in keeping with previous reports that ROS damage the DNA of Gram-negative pathogens such as Escherichia coli, Salmonella enterica, and Coxiella burnetii, with the survival of bacteria phagocytosed by macrophages being dependent upon RecBCD (46, 49, 50). Therefore, DNA is a common target of ROS produced by phagocytic immune cells for several different human pathogens.

The findings from this work build on previous studies of oxidative DNA damage and repair and enable us to understand the sequence of events that occur during the exposure of S. aureus to ROS of the respiratory burst. Of the ROS produced by neutrophils, only H_2_O_2_ can cross the membrane due to its lack of charge (14, 15, 23). In the cytoplasm, H_2_O_2_ reacts with iron in a process known as the Fenton reaction, which leads to the generation of highly reactive hydroxyl radicals (14, 15, 20, 23). These can damage DNA as well as the pool of nucleotides, leading to various types of lesions (14, 15, 20, 23). Based on our findings, physiological concentrations of ROS produced by neutrophils lead to DNA DSBs in S. aureus despite the numerous antioxidant defenses of this pathogen (21, 25, 27). While some bacteria can engage in nonhomologous end joining, most DSBs are repaired via homologous recombination (31, 33, 36). RexAB processes the broken ends to produce single-stranded DNA to which RecA binds (1, 31, 33, 36). The resulting RecA nucleoprotein filament triggers the SOS response by initiating the autocleavage of the LexA transcriptional repressor (1, 22, 51). As shown here, DNA damage processed by RexAB results in the expression of *recA*, leading to homologous recombination and survival of the bacterium. The SOS response also leads to the expression of the low-fidelity DNA polymerase UmuC, which leads to a transient increase in the mutation rate (21, 22). Previous work from our group has shown that mutagenesis due to SOS induction as a consequence of oxidative stress leads to the acquisition of mutations conferring antibiotic resistance and the small-colony phenotype associated with chronic infection and resistance to neutrophil-mediated killing (26, 37). Therefore, the processing of DNA DSBs by RexAB not only promotes the survival of bacteria exposed to ROS produced by the respiratory burst but also may promote the emergence of mutants that are more resistant to neutrophil-mediated killing by triggering the SOS response. However, this remains to be tested.

In addition to S. aureus, the RexAB system was also demonstrated to be important for the survival of the infective endocarditis pathogens E. faecalis and S. gordonii. However, while neutrophils in blood caused DNA DSBs in these pathogens, this did not appear to be due to ROS because the inhibition of NADPH oxidase with DPI had no effect on the survival of the wild type or the *rexBA* mutants. Neutrophils employ several different antibacterial elements to kill invading pathogens, including reactive oxygen and nitrogen species, proteases, and antimicrobial peptides. Several studies have indicated that bacteria vary in their susceptibility to each of these microbicides. In agreement with our findings, Standish and Weiser showed that S. aureus but not Streptococcus pneumoniae was killed by ROS (52). However, while S. aureus can grow in the presence of nitric oxide, the replication of several other pathogens, including Pseudomonas aeruginosa and Streptococcus pyogenes, is inhibited (53). Since reactive nitrogen species can cause DNA damage, this may provide an explanation for the finding that mutants of E. faecalis and S. gordonii lacking RexAB are more susceptible to killing by neutrophils in which the respiratory burst is blocked.

While the consequences of RexAB-mediated processing of DNA DSBs are relatively predictable for S. aureus, they are less so for E. faecalis and S. gordonii. This is particularly the case for *Streptococcus* since this genus lacks the LexA repressor that is central to the control of the SOS response (54–56). However, S. gordonii encodes RecA, which promotes survival during exposure to UV light, and there is evidence that the role of LexA is fulfilled by HdiR in at least some streptococci and Lactococcus lactis (56–59). For example, Streptococcus uberis has been found to encode a UmuC error-prone DNA polymerase that appears to be regulated by HdiR and induced in response to UV-mediated DNA damage, so it is possible that RexAB-mediated processing of DNA DSBs leads to the induction of an SOS-like response in S. gordonii, including homologous recombination and mutagenic DNA repair (56, 59).

In contrast to S. gordonii, E. faecalis encodes both RecA and LexA and appears to have a DNA damage-inducible UmuC polymerase, suggesting a SOS response similar to that described above for S. aureus (60–63). However, as for S. aureus, it remains to be seen whether neutrophil-mediated DNA damage leads to an increase in the mutation rate in either S. gordonii or E. faecalis.

In addition to providing protection from oxidative damage caused by neutrophils, we have previously shown that RexAB provides S. aureus with tolerance to the combination antibiotic co-trimoxazole (38). That work showed that DNA damage was partly due to endogenous oxidative stress that occurred during exposure to the combination antibiotic, in addition to direct damage to DNA caused by thymidine limitation (38). The data presented here show that the loss of RexAB also sensitizes S. aureus to ciprofloxacin, even though the JE2 strain is resistant. In addition, there is growing evidence that multiple classes of antibiotics cause endogenous ROS production in S. aureus, suggesting that RexAB may provide an important defense against damage caused by both host defenses and multiple classes of antibiotics, the two key threats to staphylococcal survival in the host. Further studies are needed to test this, but the identification of RexAB as being important for staphylococcal survival during exposure to both neutrophils and at least two antibiotics makes this complex a potential target for novel therapeutics, particularly as the lack of RexAB homologues in eukaryotes reduces the likelihood of host toxicity (64).

Inhibitors of AddAB and RecBCD have been previously reported in the literature, but problems include limited *in vivo* stability, poor oral bioavailability, and a suboptimal mechanism of action (65–68). However, Amundsen et al. identified several small-molecule inhibitors of Helicobacter pylori AddAB and E. coli RecBCD, in particular ML328 (69), indicating that the development of stable, potent inhibitors is possible. More recently, a derivative of ML328 (IMP-1700) was found to be capable of potentiating antibiotic activity such that a resistant S. aureus strain was sensitized to ciprofloxacin (70). However, the ability of IMP-1700 to inhibit AddAB/RecBCD activity under *in vivo* conditions remains to be determined. Further work in this area may lead to broad-spectrum therapeutics that promote bacterial susceptibility to both host defenses and antibiotics as well as inhibiting the induction of the mutagenic SOS response associated with the acquisition of drug resistance and host adaptation (37).

In summary, staphylococcal, streptococcal, and enterococcal DNA is damaged by the host immune system, leading to DNA DSBs that are lethal if not repaired by RexAB. These findings suggest that the RexAB complex is a potentially viable target for novel therapeutics, capable of sensitizing Gram-positive pathogens to neutrophil-mediated killing and blocking the SOS response associated with the emergence of drug resistance.

## MATERIALS AND METHODS

### Bacterial strains and culture conditions.

The bacterial strains used in this study are listed in Table 1. S. aureus was cultured in tryptic soy broth (TSB) to stationary phase (18 h) at 37°C with shaking (180 rpm). S. gordonii and E. faecalis were grown in Todd-Hewitt broth (THB) supplemented with 1% (wt/vol) yeast extract (THB-Y) at 37°C, statically in 5% CO_2_. E. coli was grown in lysogeny broth (LB) or Terrific broth (TB) (1.2% [wt/vol] tryptone, 2.4% [wt/vol] yeast extract, 0.5% glycerol, 0.17 M KH_2_PO_4_, 0.72 M K_2_HPO_4_) for protein expression at 37°C with shaking (180 rpm). Media were supplemented with antibiotics as required. When appropriate, bacteria were grown on Columbia blood agar (CBA) made with 5% defibrinated sheep blood.

**TABLE 1:** Bacterial strains used in this study

Strain	Description^*a*^	Reference or source
Staphylococcus aureus		
SH1000	*rsbU^+^* derivative of laboratory strain 8325-4	80
SH1000 *rexB*::Tn	SH1000 with a *bursa aurealis* transposon insertion in *rexB*; Ery^r^	38
SH1000 *rexB*::Tn p*itet* empty	SH1000 with a *bursa aurealis* transposon insertion in *rexB* with the integrated p*itet* empty plasmid; Ery^r^	38
SH1000 *rexB*::Tn p*itet rexAB*	SH1000 with a *bursa aurealis* transposon insertion in *rexB* with integrated p*itet* with AHT-inducible *rexB*; Ery^r^	38
JE2	Derivative of CA-MRSA USA300 LAC, cured of plasmids	24
JE2 *rexB*::Tn	JE2 with a *bursa aurealis* transposon insertion in *rexB*; Ery^r^	24
JE2 *rexB*::Tn p*itet* empty	JE2 with a *bursa aurealis* transposon insertion in *rexB* with the integrated p*itet* empty plasmid; Ery^r^	38
JE2 *rexB*::Tn p*itet rexAB*	JE2 with a *bursa aurealis* transposon insertion in *rexB* with integrated p*itet* with AHT-inducible *rexB*; Ery^r^	38
JE2 pCN34 *PrecA-gfp*	JE2 containing pCN34 with *gfp* under the control of the *recA* promoter; Kan^r^	38
JE2 *rexB*::Tn pCN34 *PrecA-gfp*	JE2 *rexB*::Tn containing pCN34 with *gfp* under the control of the *recA* promoter; Kan^r^	38

Streptococcus gordonii		
DL1 (Challis)	Wild type	81
DL1 Δ*rexBA*	DL1 with the *rexA* and *rexB* genes deleted	This study

Enterococcus faecalis		
CK111(pCF10-101)	Conjugative donor strain with pCF10-101	76
OG1X	Gelatinase deficient	82
OG1X Δ*rexBA*	OG1X with the *rexA* and *rexB* genes deleted	This study

Escherichia coli		
EC1000	Cloning host that provides RepA in *trans*	83
SoluBL21(DE3)	Derivative of BL21(DE3) for the expression of challenging proteins	Genlantis

a CA-MRSA, community-acquired methicillin-resistant S. aureus.

### P*recA*-*gfp* fluorescent reporter assay.

As detailed previously (38), promoter-reporter gene constructs in the JE2 background were used to directly assess the expression of *recA*. Antibiotic 2-fold dilutions were made in flat-bottomed black-walled 96-well plates containing TSB and kanamycin (90 μg ml^−1^) and inoculated with a 1/10 dilution of a stationary-phase culture of the reporter strains. Plates were placed into an Infinite M200-Pro microplate reader (Tecan), where cultures were grown for 17 h at 37°C (700 rpm), and both the absorbance at 600 nm (optical density at 600 nm [OD_600_]) and GFP relative fluorescence units (RFU) were measured every 30 min.

OD_600_ data and RFU data were normalized to values for the no-antibiotic controls. To account for differences in cell density, RFU values were normalized by the OD_600_ data at each time point.

### Neutrophil phagocytosis and measurement of DNA damage.

Whole human blood (15 ml) was collected from individual healthy donors in EDTA-treated tubes (BD Biosciences) and layered over 20 ml of room-temperature Polymorph prep (Alere Limited) before centrifugation at 500 × *g* for 45 to 60 min (brake off, 30°C) until a clear separation of red blood cells (RBCs), peripheral blood mononuclear cells (PBMCs), and polymorphonuclear leukocytes (PMNs) (or neutrophils) was seen. The PBMCs were discarded, and the PMNs were transferred to a fresh centrifuge tube. Hanks’ balanced salt solution (HBSS) was added to the PMNs to a total volume of 50 ml, and cells were pelleted at 500 × *g* for 10 min (brake off, 30°C). The cells were resuspended in 3 ml of HBSS, counted using a hemocytometer, and adjusted to 5 × 10^6^ cells ml^−1^ in HBSS containing 10% human serum, 0.1 mM calcium, and 0.1 mM magnesium. To the neutrophil suspension, 5 × 10^6^ cells ml^−1^ of bacteria (stationary- or exponential-phase TRITC-stained bacteria) were added. The bacterium-neutrophil suspension was then incubated at 37°C with tumbling. At each time point (0.5, 1, 2, and 3 h), 100 to 150 μl was taken and resuspended in 4% paraformaldehyde (PFA) in PBS for a minimum of 1 h. Before analysis by flow cytometry, samples were washed and resuspended in PBS. Samples were analyzed on a FACSAria or LSRFortessa flow cytometer (BD Biosciences), and at least 100,000 events were captured, except for bacterium-only samples, where at least 50,000 events were captured. Green fluorescence (GFP positive bacteria) was detected at 530 (30) nm, and TRITC labeling was detected at 586 nm (16) nm. Full gating strategies are detailed in Fig. S6 and Fig. S7 in the supplemental material. Measurements of phagocytosis include both ingested bacteria and those bound to the surface of the neutrophil, based on that of Surewaard et al. (39).

### Determination of MICs.

MICs were determined using a serial broth dilution protocol as described previously (71). Bacteria were diluted to 1 × 10^5^ CFU ml^−1^ and incubated in flat-bottomed 96-well plates with a range of antibiotic concentrations for 17 h at 37°C under static conditions (aerobic, anaerobic, or 5% CO_2_). The MIC was defined as the lowest concentration at which no growth was observed.

### Whole-blood and hydrogen peroxide survival assays.

Bacteria were washed twice with PBS and adjusted to 10^6^ CFU ml^−1^ in HBSS, and 10^4^ CFU (10 μl) were used to inoculate 90 μl of freshly donated human blood (collected in EDTA-treated tubes; BD Biosciences) or freshly diluted H_2_O_2_ (10 mM in PBS) in 96-well plates. Ethical approval for drawing and using human blood was obtained from the Regional Ethics Committee and the Imperial NHS Trust Tissue Bank (REC Wales approval no. 12/WA/0196 and ICHTB HTA license no. 12275). In some assays, blood was pretreated for 10 min with diphenyleneiodonium (DPI) (50 μM) or an equivalent volume of DMSO as a solvent control. After 6 h of incubation, bacterial survival was determined by CFU counts in blood-bacterium mixtures on CBA plates. For H_2_O_2_ assays, survival was measured after 1 h at 37°C (static) in the dark. Survival for both assays was calculated as a percentage of the number of bacteria in the starting inoculum.

### Neutrophil survival assay.

Neutrophils were adjusted to 5 × 10^6^ cells ml^−1^ in HBSS containing 10% human serum, 0.1 mM calcium, and 0.1 mM magnesium. Stationary-phase bacterial cultures were washed in PBS, and 1 × 10^6^ CFU were added to the neutrophil suspension (multiplicity of infection [MOI] of 1:5) to a total volume of 1 ml. Neutrophils were treated for 10 min prior to the addition of bacteria with either DPI (50 μM) or an equivalent volume of DMSO (solvent control), as needed. The bacterium-neutrophil suspension was subsequently incubated at 37°C with tumbling. At relevant time points (0.5, 1, 2, and 3 h), 50 μl of the suspension was transferred to a 96-well plate and serially diluted 10-fold in PBS up to a 10^−3^ dilution. All dilutions (including neat) were then plated onto CBA and incubated for 24 h at 37°C before counting. Survival was calculated as a percentage of the number of bacteria in the starting inoculum.

### Murine systemic infection model.

Animal work was conducted in accordance with the Animals (Scientific Procedures) Act 1986 outlined by United Kingdom Home Office regulations. Work was approved by the United Kingdom Home Office after ethical approval by the Imperial College Animal Welfare and Ethical Review Body (AWERB). Six- to eight-week-old female C57BL/6 mice (Charles River) were infected via the intraperitoneal route with the wild type or the *rexB*::Tn mutant in the JE2 and SH1000 backgrounds. Stationary-phase bacterial cultures were washed twice with PBS and adjusted to 10^7^ CFU ml^−1^. Subsequently, 400 μl (4 × 10^6^ CFU) of the washed bacterial suspensions was injected into the peritoneal cavity of each mouse (5 mice for each strain; 20 in total). After 6 h, the mice were humanely sacrificed by cervical dislocation, and death was confirmed by severing the femoral artery. The peritoneal cavity was washed with PBS to release the bacteria, and CFU counts were determined by plating onto tryptic soy agar (TSA). Sample size was determined prior to the experiment using power analysis based on *in vitro* data (72). Tubes containing the bacterial suspensions were blinded before the start of the experiment. Mice were randomly allocated to group cages, and each group was randomly allocated to a treatment. According to Home Office regulations, any animals that displayed two or more of the following symptoms were humanely killed using a schedule 1 method and excluded from the study: shivering, hunched posture, reduced movement, cyanosis, circling, or difficulty breathing.

### Murine skin infection model.

Six- to eight-week-old mice were subcutaneously infected with 2 × 10^6^ CFU of exponential-phase cultures of S. aureus as previously described (73). Skin infection animal work was performed according to the *Guide for the Care and Use of Laboratory Animals* of the National Institutes of Health (74), the Animal Welfare Act, and U.S. Federal law. The protocol was approved by the Animal Care and Use Committee of Rutgers New Jersey Medical School.

### Construction of Δ*rexBA* mutants of E. faecalis and S. gordonii.

A Δ*rexBA* mutant was generated in S. gordonii by in-frame allelic replacement with the erythromycin resistance determinant *ermAM*. Flanking regions directly upstream and downstream of the *rexBA* operon were amplified by PCR from S. gordonii DL1 genomic DNA with primer pairs Sg.rexAB.F1/Sg.rexAB.R1 and Sg.rexAB.F2/Sg.rexAB.R2 (Table 2), respectively, while the *ermAM* cassette was amplified from plasmid pVA838 (75) using primer pair ermAM.SgF/ermAM.SgR (Table 2). The resulting amplimers were then joined via 20-bp overlapping regions by stitch PCR using primers Sg.rexAB.F1/Sg.rexAB.R2 and transformed into S. gordonii. Erythromycin-resistant transformants were confirmed by sequencing, and the strain was designated UB3018.

**TABLE 2:** Primers used in this study

Oligonucleotide	Sequence (5′–3′)^*a*^
rexB-F BamHI	CCAGGATCCGATGACATTACATGCTTATTTAGG
rexA-R SalI	GCGGTCGACCTATAGTTGCAATGTACC
StrepII rexAB SDM-F	ACAATTCCAGAGAAACCACAAGGCGTGATTTGGACTGACGCGCAATGGC
StrepII rexAB SDM-R	AGCGCTTTTTTCGAACTGCGGGTGGCTCCACATCTATTGCTCACCCCC
Thr rexAB SDM-F	CCCGCAGTTCGAAAAAAGCGCTGGCCTGGTGCCGCGCGGCAGCGGCACAATTCCAGAGAAACCACAAGG
Thr rexAB SDM-R	CCTTGTGGTTTCTCTGGAATTGTGCCGCTGCCGCGCGGCACCAGGCCAGCGCTTTTTTCGAACTGCGGG
Sg.rexAB.F1	CAGCAGGACAAGGGAAGTG
Sg.rexAB.R1	TTTTGTTCATAGCCTGCCTTTTCCTCTAG
Sg.rexAB.F2	AGGTCCCTAGAAGGAAGCATCTGAGTTG
Sg.rexAB.R2	CGTCGAGCACTAGTCTCG
ermAM.SgF	AAGGCAGGCTATGAACAAAAATATAAAATATTCTCA
ermAM.SgR	ATGCTTCCTTCTAGGGACCTCTTTAGCTCC
Ef.rexAB.pheF1	TGACGTCGACGCGT*CTGCAG*GATCGCTAAAACGCTAGAAGC
Ef.rexAB.pheR1	TAAAATCTACCTGCACACTCATGGTTTCAC
Ef.rexAB.pheF2	GAGTGTGCAGGTAGATTTTATAAAGTAGAAAAAATTAGAAG
Ef.rexAB.pheR2	GCTTAGCATG*CCATGG*TCTTAATACTTCGGTGATTGG

a Underlined regions indicate base pair overlaps for stitch PCR, and italicized regions indicate restriction endonuclease sites for ligation into pCJK47.

A *rexBA* mutant was generated in E. faecalis by markerless exchange using a 2-step homologous recombination approach, as previously described (76). In brief, flanking regions directly upstream and downstream of the *rexBA* operon were amplified by PCR from E. faecalis OG1X genomic DNA with primer pairs Ef.rexAB.pheF1/Ef.rexAB.pheR1 and Ef.rexAB.pheF2/Ef.rexAB.pheR2 (Table 2), respectively, and then joined via 20-bp overlapping regions by stitch PCR using primer pair Ef.rexAB.pheF1/Ef.rexAB.pheR2. The resultant amplimer was cloned into donor plasmid pCJK47 (76) via the unique restriction sites PstI and NcoI to generate pCJK47-*rexAB*, propagated in E. coli EC1000, and then introduced into conjugative donor strain E. faecalis CK111(pCF10-101) by electroporation. Plasmid pCJK47-*rexAB* was transferred to E. faecalis OG1X by conjugation. Transconjugants carrying the integrated plasmid were confirmed by colony PCR, before counterselection based on the P-pheS* marker was used to identify secondary recombinants in which the integrated plasmid had been excised and lost, leaving the desired Δ*rexBA* allele. This was confirmed by sequencing, and the strain was designated UB2948.

### Construction of an S. aureus RexAB expression vector.

Cloning of the *rexA* and *rexB* genes from S. aureus was achieved by PCR from wild-type genomic JE2 DNA using the rexB-F BamHI and rexA-R SalI primers listed in Table 2, which allowed the amplification of the *rexBA* operon immediately flanked by suitable restriction endonuclease recognition sequences (BamHI and SalI). The *rexA* and *rexB* genes were inserted into the pET28b^+^ vector (Novagen) using standard cloning techniques, and site-directed mutagenesis (SDM) was performed to insert Strep-tag II and a thrombin site in front of the *rexA* gene. This enabled RexA and RexB proteins to be detected individually via an N-terminal His_6_ tag for RexB (His_6_ from the pET28b^+^ vector) and N-terminal Strep-tag II for RexA. PCR primers for SDM are listed in Table 2. DNA sequencing was used on the pET28b^+^ *rexBA* expression plasmid to confirm that the sequences of the entire *rexA* and *rexB* genes, tags, and promoter regions were as expected.

### Expression and purification of recombinant S. aureus RexAB.

Cells from single colonies of E. coli SoluBL21(DE3), freshly transformed with the pET28b^+^ *rexAB* expression plasmid coding for N-terminally Strep-tag II-tagged RexA and N-terminally His_6_-tagged RexB, were used to inoculate a starter culture grown overnight in LB supplemented with 50 μg ml^−1^ of kanamycin. The starter culture was diluted to an OD_600_ of 0.05 into 4 liters of TB containing 50 μg ml^−1^ of kanamycin. Cells were grown at 37°C with shaking at 180 rpm until an OD_600_ of 0.5 was reached, prior to induction with 1 mM isopropyl-β-d-thiogalactoside (IPTG). Following induction, the temperature was reduced to 20°C, and cultures were further incubated for 20 h. Cells were harvested by centrifugation at >10,000 × *g* at 4°C for 30 min, and pellets were resuspended in 100 ml of a solution containing 50 mM Tris and 150 mM NaCl (pH 7.5).

For protein purification, cells were disrupted by sonication, and cell debris was cleared by centrifugation at 32,000 × *g* at 4°C for 30 min. The resulting supernatant was added to 5 ml of Chelating Sepharose Fast Flow resin (GE Healthcare), which had been loaded with 0.1 M NiCl_2_ and equilibrated with 100 ml of a solution containing 50 mM Tris, 150 mM NaCl, and 20 mM imidazole (pH 7.5). The supernatant-resin mixture was left at 4°C overnight with gentle stirring to optimize the binding of the His-tagged protein to the nickel-charged resin. Next, the mixture was washed nine times with 40 ml of a solution containing 50 mM Tris, 150 mM NaCl, and 20 mM imidazole (pH 7.5) and once with 20 ml of a solution containing 50 mM Tris, 150 mM NaCl, and 70 mM imidazole (pH 7.5). His-tagged protein was eluted with a solution containing 50 mM Tris, 150 mM NaCl, and 150 mM imidazole (pH 7.5); each 10-ml fraction was tested with Bradford reagent (Bio-Rad) for protein content until no more protein could be detected. Fractions containing protein were pooled, buffer exchanged, and concentrated using an Amicon 100-kDa-cutoff concentrator (Merck Millipore); RexAB is >250 kDa. The concentrating device was centrifuged at 2,000 × *g* (4°C), and the protein solution was exchanged into a solution containing 50 mM Tris and 150 mM NaCl (pH 7.5) by four serial concentration and redilution steps. The total protein concentration was quantified using the Pierce bicinchoninic acid (BCA) protein assay kit (Thermo Fisher Scientific) according to the manufacturer’s instructions, and the presence of intact recombinant RexAB was confirmed via SDS-PAGE and Western blot analysis.

### Nuclease and helicase activity assays.

The nuclease and helicase activities of RexAB were measured to confirm the AddAB-like activity in our recombinant RexAB protein. Staphylococcal DNA was amplified from the JE2 whole genome by colony PCR using the primer pair Chi control F (5′-TCAGTGAATTAGATGATTCGC-3′) and Chi control R (5′-TTCATACGTATGAATGTTATTTGC-3′), where the amplicon lacked a Chi site region to be used as the DNA substrate in these assays.

Reactions were set up with either nuclease assay buffer (25 mM Tris-acetate [pH 7.5], 2 mM Mg acetate, 1 mM dithiothreitol [DTT]) or helicase assay buffer (25 mM Tris-acetate [pH 7.5], 0.25 mM Mg acetate, 1 mM DTT), along with 5 ng μl^−1^ of DNA, 1 mM ATP or an equivalent volume of nuclease-free water, and either 20 nM or 50 nM recombinant RexAB for the nuclease and helicase assays, respectively. Additionally, for the helicase assay, 2 μM SSB protein was added to each sample.

Samples were incubated statically at 37°C, and at 0, 5, 15, 30, 60, and 120 min, 5 μl was removed and pipetted into 20 μl of STEB buffer (40% [wt/vol] sucrose, 100 mM Tris-HCl, 10 mM EDTA, 0.5 mg/ml bromophenol blue [pH 8]) to stop the reaction. Twenty microliters of chloroform-isoamyl alcohol (24:1) was added to each tube, vortexed for 10 s, and centrifuged for 2 min at 17,000 × *g* to remove the protein and any compounds used for inhibition. The aqueous (upper blue) phase was loaded onto a 1% (wt/vol) agarose gel prepared in Tris-borate-EDTA (TBE) buffer, and electrophoresis was carried out at 85 V for 1 h. The gels were subsequently stained with SYBR Safe DNA gel stain (Invitrogen) at a 1/10,000 dilution in TBE buffer for 2 h with rocking and visualized using a Gel Doc EZ imager (Bio-Rad). The band intensity was quantified using ImageJ software. For nuclease activity, values were normalized to those of the no-ATP controls at 0 h. For helicase activity, values were normalized to those of an ssDNA control that lacked the RexAB protein, which had been heated at 95°C for 2 min to denature the dsDNA and allow the SSB protein to bind and stabilize the two ssDNA strands.

### Measurement of bacterial growth.

To measure the growth of S. aureus, bacterial cultures were first grown to stationary phase in TSB at 37°C (180 rpm) and then inoculated at a 1/50 dilution for growth curves or a 1/10 dilution for growth inhibition assays (supplementary) into a flat-bottomed 96-well plate (200-μl total volume) and placed into a POLARstar Omega plate reader (BMG Labtech). Bacteria were grown for 17 h at 37°C (700 rpm), and the absorbance at 600 nm was measured every 30 min.

### Hemolytic activity.

The hemolytic activity of culture supernatants was determined as described previously (77). Briefly, stationary-phase S. aureus cultures were pelleted for 5 min at 17,000 × *g*, and 400 μl of the supernatant was pipetted into microcentrifuge tubes. An equal volume of 2% defibrinated sheep blood in PBS was added, and the mixture was incubated statically for 1 h at 37°C. Fresh TSB containing 2% defibrinated sheep blood was used as a negative control. After incubation, the samples were centrifuged for 5 min at 500 × *g*, and 200 μl of the supernatant was transferred into a flat-bottomed 96-well plate. The absorbance of released hemoglobin was measured at 540 nm using a POLARstar Omega plate reader (BMG Labtech). Percent hemolysis was calculated relative to the wild-type control.

### Staphyloxanthin extraction and quantification.

To extract and quantify staphyloxanthin, S. aureus stationary-phase cultures grown in TSB were harvested by centrifugation at 17,000 × *g* for 2 min. Staphyloxanthin was extracted by incubating the culture in methanol at 42°C for 30 min. Cells were pelleted by centrifugation at 17,000 × *g* for 2 min, and 100 μl of the supernatant was transferred into a flat-bottomed 96-well plate. The released staphyloxanthin was quantified by measuring the absorbance at 462 nm using a POLARstar Omega plate reader (BMG Labtech).

### Catalase activity.

To determine the level of catalase activity, cultures grown overnight were washed three times in PBS, and 10^7^ CFU were inoculated into 1 ml of 100 μM H_2_O_2_ (diluted in PBS). Samples were incubated at 37°C, protected from light, for 15 min. Two hundred microliters of the sample was centrifuged at 17,000 × *g* for 3 min, and 20 μl of the supernatant was added to a 96-well microtiter plate. PBS containing no bacteria was used as a negative control.

Catalase activity was determined indirectly by measuring the concentration of H_2_O_2_ over time using the Pierce quantitative peroxide assay kit (aqueous-compatible formulation; Thermo Fisher Scientific). According to the manufacturer’s instructions, reagents A and B were mixed at a ratio of 1:100, and 200 μl was added to each sample in the 96-well plate. The plate was then incubated for 30 min at room temperature, and the absorbance was measured at 595 nm using an iMark microplate reader (Bio-Rad). The H_2_O_2_ concentration was determined using a standard curve of known concentrations (up to 1 mM).

### Computational analyses.

Multiple-sequence alignments were generated using Clustal Omega via the EMBL-EBI Web server (78). Parameters were left in their default settings with the exception of alignment “order,” which was set to consider the input order. Protein structures were predicted using the Phyre2 protein fold recognition server (84). Phyre2 structural models were viewed and manipulated using PyMOL molecular graphics system version 2.3 (Schrödinger).

### Statistical analyses.

Data are presented as the means or medians from three or more independent experiments and were analyzed by Student’s *t* test (two tailed, unpaired, and assuming equal variances), one-way analysis of variance (ANOVA), or two-way ANOVA corrected for multiple comparisons, as described in the figure legends. For each experiment, “*n*” refers to the number of independent biological replicates. CFU counts from murine experiments are presented as the values obtained from each animal, and significance was assessed using the Mann-Whitney test. A *P* value of <0.05 was considered significant between data points (GraphPad Prism 7 for Windows).
