# Supplementary material for: Staphylococcal DNA Repair Is Required for Infection
Source: mBio. 2020 Nov 17;11(6):e02288-20. doi: 10.1128/mBio.02288-20 (PMC7683395; doi:10.1128/mBio.02288-20)
Supplement: TABLE S1 [file mBio.02288-20-st001.docx]

|  | **Strain** | | | |
| --- | --- | --- | --- | --- |
| **Antibiotic** | *S. gordonii* DL1 WT | *S. gordonii* Δ*rexAB* | *E. faecalis* DL1 WT | *E. faecalis* Δ*rexAB* |
| Ciprofloxacin | 1.0 | 0.25 | 1.0 | 0.03 |
| Gentamicin | 0.5 | 0.5 | 0.5 | 0.5 |
